# Supplementary material for: Adaptive evolution of odorant receptors is associated with elaborations of social organization in ants
Source: Mol Biol Evol. 2026 Apr 17;43(5):msag103. doi: 10.1093/molbev/msag103 (PMC13166875; doi:10.1093/molbev/msag103)
Supplement: msag103_Supplementary_Data [file msag103_supplementary_data.zip › Supplementary Table S2 - PGLS.pdf]

Table S2: Results of the PGLS analysis, after correction for multiple testing.

Red shows q-values of less than 5%, orange shows q-values of less than 10%.

ORs subfamily	Average colony size	Social parasitism	Inquilinism	Workers polymorphism	Diet	Workers ovary	Workers spermatheca	Workers sterility
Total ORs	0.14512209	0.00001490	0.00000003	0.87830646	0.85170400	0.93534900	1.00000000	0.99744600
OR_9.exon	0.01190480	0.00002670	0.00000003	0.87830646	0.30158100	1.00000000	1.00000000	0.99744600
OR_A	0.72294106	0.00304436	0.01972397	0.85814551	0.30189000	0.91336400	1.00000000	0.89163100
OR_D	0.32007683	0.22164471	0.27175448	0.74541856	0.95683800	0.14780100	1.00000000	0.99744600
OR_E	0.82200192	0.05677850	0.01803762	0.74541856	0.86333900	0.98625400	0.72339500	0.89163100
OR_F	0.03187228	0.00188470	0.00055200	0.74541856	0.86333900	0.98625400	1.00000000	0.99744600
OR_G	0.56884505	0.20852422	1.00000000	0.80409492	0.98814400	1.00000000	1.00000000	0.99744600
OR_H	0.99556976	0.47499578	0.90865603	0.74541856	0.30158100	0.93534900	1.00000000	0.99744600
OR_I	0.70400129	0.46300096	1.00000000	0.74541856	0.59035000	1.00000000	0.90025800	0.99744600
OR_J	0.86109280	0.30388827	0.25695468	0.74541856	1.00000000	0.98625400	1.00000000	0.99744600
OR_L	0.20188714	0.00002670	0.00000000	0.85100253	0.90443700	0.93534900	1.00000000	0.99744600
OR_M	0.84273361	0.00002670	0.00000006	0.74541856	0.85343200	0.93534900	0.90628000	0.99744600
OR_N	0.38632040	0.07018520	0.00178027	0.98077757	0.54826000	0.91336400	1.00000000	0.99744600
OR_O	0.02534427	0.05677850	0.00000101	0.74541856	0.85343200	0.93534900	1.00000000	0.99744600
OR_P	0.29849008	0.00606160	0.00088900	0.93526994	0.91324400	0.98625400	1.00000000	0.99744600
OR_Q	0.95748658	0.72642294	1.00000000	0.74541856	0.85343200	0.93534900	1.00000000	0.99744600
OR_T	0.00872355	0.01103333	0.00023900	0.98555930	0.59035000	0.93534900	1.00000000	0.99744600
OR_U	0.56884505	0.07828524	0.05202740	0.91887268	0.61700400	0.98625400	0.84243100	0.89163100
OR_V	0.01190480	0.00002670	0.00023900	0.74541856	0.85170400	0.98625400	1.00000000	0.99744600
OR_with_partial	0.00169118	0.00002670	0.00000000	0.95007586	0.86333900	0.98625400	1.00000000	0.99744600
