# Supplementary material for: Adaptive evolution of odorant receptors is associated with elaborations of social organization in ants
Source: Mol Biol Evol. 2026 Apr 17;43(5):msag103. doi: 10.1093/molbev/msag103 (PMC13166875; doi:10.1093/molbev/msag103)
Supplement: msag103_Supplementary_Data [file msag103_supplementary_data.zip › Supplementary Table S3 - Positive selection.pdf]

All ORs

Clade	Species branch of interest	Sum of the length of all gene tree branches in the clade	Total number of positive gene tree branches in the clade	Sum of the length of gene tree branches mapped on the species branch of interest	Number of positive gene tree branches mapped on the species branch of interest	p-value	q-value	Fold change of normalized positive branches	Trait change mapped on the species branch
**Lasius-Nylanderia**	68	5667	276	370	16	0.091620296	0.11689486	0.887896592	Increased colony size
**Lasius-Nylanderia**	69			423	18	0.081515848	0.11689486	0.87372803	Worker Polymorphism (Discrete)
**Lasius-Nylanderia**	92			207	15	0.034553758	0.11689486	1.487870195	Worker Polymorphism (Continuous)
**Lasius-Nylanderia**	94			309	13	0.097889054	0.117886164	0.863831434	Diet - Omnivore to Herbivore, Worker Polymorphism (Discrete)
**Lasius-Nylanderia**	110			113	14	0.000735556	0.024611531	2.543863024	Worker Polymorphism (Continuous)
**Lasius-Nylanderia**	112			305	10	0.048421076	0.11689486	0.673200285	Diet - Omnivore to Herbivore, Worker Polymorphism (Discrete)
**Lasius-Nylanderia**	179			36	2	0.274611028	0.274611028	1.140700483	Increased colony size
**Leaf-cutter-ants**	305	9260	344	372	9	0.048342252	0.11689486	0.651256564	Worker Polymorphism (Discrete)
**Leaf-cutter-ants**	311			319	8	0.067298973	0.11689486	0.675074725	Increased colony size, Worker Polymorphism (Discrete)
**Leaf-cutter-ants**	314			294	8	0.090454767	0.11689486	0.732479038	Increased colony size, Loss of Worker Reproductive Function, Worker Polymorphism (Discrete)
**Leaf-cutter-ants**	315			325	8	0.062176311	0.11689486	0.662611807	Increased colony size, Worker Polymorphism (Discrete)
**Poneroid**	12	4827	271	274	8	0.013470676	0.098042717	0.520052792	Loss of Worker Reproductive Function
**Formica-Camponotus**	97	13301	495	563	10	0.002865778	0.032618275	0.477277213	Worker Polymorphism (Discrete)
**Formica-Camponotus**	115			353	13	0.113434837	0.123443793	0.989572782	Worker Polymorphism (Continuous)
**Formica-Camponotus**	116			642	22	0.081118866	0.11689486	0.920803046	Worker Polymorphism (Discrete)
**Formica-Camponotus**	138			285	6	0.046836174	0.11689486	0.565699096	Worker Polymorphism (Continuous)
**Formica-Camponotus**	139			358	17	0.060849178	0.11689486	1.275983297	Increased colony size, Worker Polymorphism (Continuous)
**Formica-Camponotus**	140			444	11	0.040045334	0.11689486	0.665715716	Worker Polymorphism (Discrete)
**Formica-Camponotus**	157			184	5	0.133999282	0.137721484	0.730182257	Worker Polymorphism (Continuous)
**Formica-Camponotus**	158			195	10	0.079378381	0.11689486	1.377984978	Increased colony size, Worker Polymorphism (Continuous)
**Formica-Camponotus**	160			440	20	0.061933249	0.11689486	1.221395776	Worker Polymorphism (Discrete)
**Messor**	144	6227	265	466	23	0.06765569	0.11689486	1.15977812	Increased colony size
**Messor**	145			430	18	0.098769489	0.117886164	0.983641948	Diet - Omnivore to Herbivore
**Messor**	186			325	11	0.088208717	0.11689486	0.795320755	Diet - Omnivore to Herbivore
**Pheidole**	172	9350	467	442	21	0.088283424	0.11689486	0.951243617	Loss of Worker Reproductive Function
**Pheidole**	199			470	32	0.015898819	0.098042717	1.363160053	Worker Polymorphism (Continuous)
**Pheidole**	200			487	13	0.0035263	0.032618275	0.53445251	Increased colony size
**Pheidole**	228			512	33	0.024465661	0.11689486	1.290442653	Increased colony size, Loss of Worker Reproductive Function, Worker Polymorphism (Discrete)
**Carebara**	171	8531	460	716	23	0.001330353	0.024611531	0.595740223	Loss of Worker Reproductive Function
**Carebara**	194			284	16	0.102242077	0.118217402	1.044825475	Loss of Worker Reproductive Function, Worker Polymorphism (Discrete)
**Carebara**	222			190	11	0.120870277	0.12777715	1.073695652	Worker Polymorphism (Discrete)
**Myrmica**	120	3731	245	288	24	0.042528426	0.11689486	1.269047619	Diet - Omnivorevore to Carnivore
**Leaf-cutter-ancestor**	175	11946	537	509	16	0.029218855	0.11689486	0.699278902	Diet - Omnivore to Herbivore
**Leaf-cutter-ancestor**	203			337	12	0.080724889	0.11689486	0.79213567	Diet - Omnivore to Herbivore
**Formicoid**	5	16953	981	337	21	0.085158892	0.11689486	1.076879101	
**Formicoid**	9			327	22	0.068521074	0.11689486	1.162659335	
**Formicoid**	17			252	14	0.108464326	0.121611517	0.960074754	

9-Exons only

Clade	Species branch	Sum of the length of all gene tree branches	Total number of positive gene tree branches	Sum of the length of gene tree branches mapped on the species branch of interest	Number of positive gene tree branches mapped on the species branch of interest	p-value	q-value	Fold change of normalized positive branches	Trait change mapped on the species branch
**Lasius-Nylanderia**	68			117	8	0.148437	0.192875714	0.962691629	Increased colony size
**Lasius-Nylanderia**	69			126	6	0.090181	0.187753857	0.670445956	Worker Polymorphism (Discrete)
**Lasius-Nylanderia**	92			58	9	0.012342	0.152218	2.184729064	Worker Polymorphism (Continuous)
**Lasius-Nylanderia**	94	1774	126	96	6	0.160439	0.192875714	0.879960317	Diet - Omnivore to Herbivore, Worker Polymorphism (Discrete)
**Lasius-Nylanderia**	110			40	10	0.000251	0.009287	3.51984127	Worker Polymorphism (Continuous)
**Lasius-Nylanderia**	112			100	6	0.15328	0.192875714	0.844761905	Diet - Omnivore to Herbivore, Worker Polymorphism (Discrete)
**Lasius-Nylanderia**	179			14	0	0.35509	0.35509	#NUM!	Increased colony size
**Leaf-cutter-ants**	305			138	4	0.099643	0.187753857	0.594902549	Worker Polymorphism (Discrete)
**Leaf-cutter-ants**	311	2976	145	116	3	0.102116	0.187753857	0.530796671	Increased colony size, Worker Polymorphism (Discrete)
**Leaf-cutter-ants**	314			97	5	0.181793	0.192875714	1.057945254	Increased colony size, Loss of Worker Reproductive Function, Worker Polymorphism (Discrete)
**Leaf-cutter-ants**	315			110	5	0.180337	0.192875714	0.932915361	Increased colony size, Worker Polymorphism (Discrete)
**Poneroid**	12	1547	114	72	4	0.168141	0.192875714	0.753898635	Loss of Worker Reproductive Function
**Formica-Camponotus**	97			207	6	0.076449	0.187753857	0.636832212	Worker Polymorphism (Discrete)
**Formica-Camponotus**	115			145	7	0.153032	0.192875714	1.060655028	Worker Polymorphism (Continuous)
**Formica-Camponotus**	116			231	11	0.12372	0.192875714	1.046224347	Worker Polymorphism (Discrete)
**Formica-Camponotus**	138			130	2	0.043386	0.187753857	0.338010943	Worker Polymorphism (Continuous)
**Formica-Camponotus**	139	5251	239	148	9	0.09536	0.187753857	1.336056768	Increased colony size, Worker Polymorphism (Continuous)
**Formica-Camponotus**	140			201	8	0.133534	0.192875714	0.874456171	Worker Polymorphism (Discrete)
**Formica-Camponotus**	157			98	5	0.176003	0.192875714	1.120954658	Worker Polymorphism (Continuous)
**Formica-Camponotus**	158			87	7	0.056573	0.187753857	1.76775838	Increased colony size, Worker Polymorphism (Continuous)
**Formica-Camponotus**	160			189	11	0.089983	0.187753857	1.278718647	Worker Polymorphism (Discrete)
**Messor**	144			161	8	0.14779	0.192875714	1.041603188	Increased colony size
**Messor**	145	2222	106	146	9	0.10432	0.187753857	1.292194365	Diet - Omnivore to Herbivore
**Messor**	186			122	7	0.140003	0.192875714	1.202752861	Diet - Omnivore to Herbivore
**Pheidole**	172			134	6	0.145517	0.192875714	0.820008068	Loss of Worker Reproductive Function
**Pheidole**	199	3388	185	184	15	0.033401	0.187753857	1.492949471	Worker Polymorphism (Continuous)
**Pheidole**	200			179	4	0.018186	0.1682205	0.409240525	Increased colony size
**Pheidole**	228			190	12	0.106563	0.187753857	1.156642959	Increased colony size, Loss of Worker Reproductive Function, Worker Polymorphism (Discrete)
**Carebara**	171			249	5	0.003247	0.0600695	0.367359007	Loss of Worker Reproductive Function
**Carebara**	194	2982	163	79	4	0.200383	0.205949194	0.92630271	Loss of Worker Reproductive Function, Worker Polymorphism (Discrete)
**Carebara**	222			65	1	0.095583	0.187753857	0.281453516	Worker Polymorphism (Discrete)
**Myrmica**	120	1461	102	117	12	0.050246	0.187753857	1.46907994	Diet - Omnivorevore to Carnivore
**Leaf-cutter-ancestor**	175	3762	196	157	5	0.082799	0.187753857	0.611269986	Diet - Omnivore to Herbivore
**Leaf-cutter-ancestor**	203			97	5	0.18245	0.192875714	0.989375131	Diet - Omnivore to Herbivore
**Formicoid**	5			112	9	0.098298	0.187753857	1.320283883	
**Formicoid**	9	5767	351	108	10	0.057171	0.187753857	1.521314762	
**Formicoid**	17			79	7	0.097779	0.187753857	1.455840456	

Without 9-Exons

Clade	Species branch	Sum of the length of all gene tree branches	Total number of positive gene tree branches	Sum of the length of gene tree branches mapped on the species branch of interest	Number of positive gene tree branches mapped on the species branch of interest	p-value	q-value	Fold change of normalized positive branches	Trait change mapped on the species branch
**Lasius-Nylanderia**	68	3893	150	253	8	0.121234	0.161843103	0.820658762	Increased colony size
**Lasius-Nylanderia**	69			299	12	0.120345	0.161843103	1.041605351	Worker Polymorphism (Discrete)
**Lasius-Nylanderia**	92			150	6	0.166553	0.176070314	1.038133333	Worker Polymorphism (Continuous)
**Lasius-Nylanderia**	94			214	7	0.139209	0.1716911	0.84894081	Diet - Omnivore to Herbivore, Worker Polymorphism (Discrete)
**Lasius-Nylanderia**	110			72	4	0.157994	0.174522471	1.441851852	Worker Polymorphism (Continuous)
**Lasius-Nylanderia**	112			205	4	0.056159	0.161843103	0.506406504	Diet - Omnivore to Herbivore, Worker Polymorphism (Discrete)
**Lasius-Nylanderia**	179			21	2	0.148012	0.174522471	2.471746032	Increased colony size
**Leaf-cutter-ants**	305	6284	199	227	5	0.120841	0.161843103	0.695548226	Worker Polymorphism (Discrete)
**Leaf-cutter-ants**	311			199	5	0.154353	0.174522471	0.793414308	Increased colony size, Worker Polymorphism (Discrete)
**Leaf-cutter-ants**	314			197	3	0.07594	0.161843103	0.480881565	Increased colony size, Loss of Worker Reproductive Function, Worker Polymorphism (Discrete)
**Leaf-cutter-ants**	315			213	3	0.056885	0.161843103	0.444759006	Increased colony size, Worker Polymorphism (Discrete)
**Poneroid**	12	3280	157	204	4	0.018213	0.161843103	0.409641564	Loss of Worker Reproductive Function
**Formica-Camponotus**	97	8050	256	359	4	0.006512	0.161843103	0.350365599	Worker Polymorphism (Discrete)
**Formica-Camponotus**	115			207	6	0.160372	0.174522471	0.911458333	Worker Polymorphism (Continuous)
**Formica-Camponotus**	116			414	11	0.100814	0.161843103	0.835503472	Worker Polymorphism (Discrete)
**Formica-Camponotus**	138			153	4	0.183086	0.188171722	0.822099673	Worker Polymorphism (Continuous)
**Formica-Camponotus**	139			210	8	0.12685	0.161843103	1.197916667	Increased colony size, Worker Polymorphism (Continuous)
**Formica-Camponotus**	140			240	3	0.033219	0.161843103	0.393066406	Worker Polymorphism (Discrete)
**Formica-Camponotus**	157			82	0	0.069689	0.161843103	#NUM!	Worker Polymorphism (Continuous)
**Formica-Camponotus**	158			107	3	0.222739	0.222739	0.881644276	Increased colony size, Worker Polymorphism (Continuous)
**Formica-Camponotus**	160			249	9	0.12667	0.161843103	1.13657756	Worker Polymorphism (Discrete)
**Messor**	144	4005	159	306	15	0.077427	0.161843103	1.234739179	Increased colony size
**Messor**	145			285	9	0.103864	0.161843103	0.795431976	Diet - Omnivore to Herbivore
**Messor**	186			203	4	0.051556	0.161843103	0.496328655	Diet - Omnivore to Herbivore
**Pheidole**	172	5962	282	306	15	0.10682	0.161843103	1.036364901	Loss of Worker Reproductive Function
**Pheidole**	199			287	17	0.065646	0.161843103	1.252304347	Worker Polymorphism (Continuous)
**Pheidole**	200			308	9	0.03537	0.161843103	0.617781155	Increased colony size
**Pheidole**	228			323	21	0.031739	0.161843103	1.374547131	Increased colony size, Loss of Worker Reproductive Function, Worker Polymorphism (Discrete)
**Carebara**	171	5549	297	467	18	0.028664	0.161843103	0.720134969	Loss of Worker Reproductive Function
**Carebara**	194			208	12	0.115978	0.161843103	1.077894328	Loss of Worker Reproductive Function, Worker Polymorphism (Discrete)
**Carebara**	222			125	10	0.06103	0.161843103	1.494680135	Worker Polymorphism (Discrete)
**Myrmica**	120	2270	143	171	12	0.114544	0.161843103	1.113973746	Diet - Omnivorevore to Carnivore
**Leaf-cutter-ancestor**	175	8184	341	353	11	0.070807	0.161843103	0.747875354	Diet - Omnivore to Herbivore
**Leaf-cutter-ancestor**	203			241	7	0.088141	0.161843103	0.697095436	Diet - Omnivore to Herbivore
**Formicoid**	5	11186	630	225	12	0.116379	0.161843103	0.946962963	
**Formicoid**	9			219	12	0.118187	0.161843103	0.972907154	
**Formicoid**	17			173	7	0.096769	0.161843103	0.718432884	
