# Supplementary material for: Adaptive evolution of odorant receptors is associated with elaborations of social organization in ants
Source: Mol Biol Evol. 2026 Apr 17;43(5):msag103. doi: 10.1093/molbev/msag103 (PMC13166875; doi:10.1093/molbev/msag103)
Supplement: msag103_Supplementary_Data [file msag103_supplementary_data.zip › Supplementary_figures.pdf]

Supplementary figures

Figures S1 to S4: Ancestral trait reconstructions made with the R package ape. For figures S2 to S4, circles show the probability for each trait to be the ancestral state.

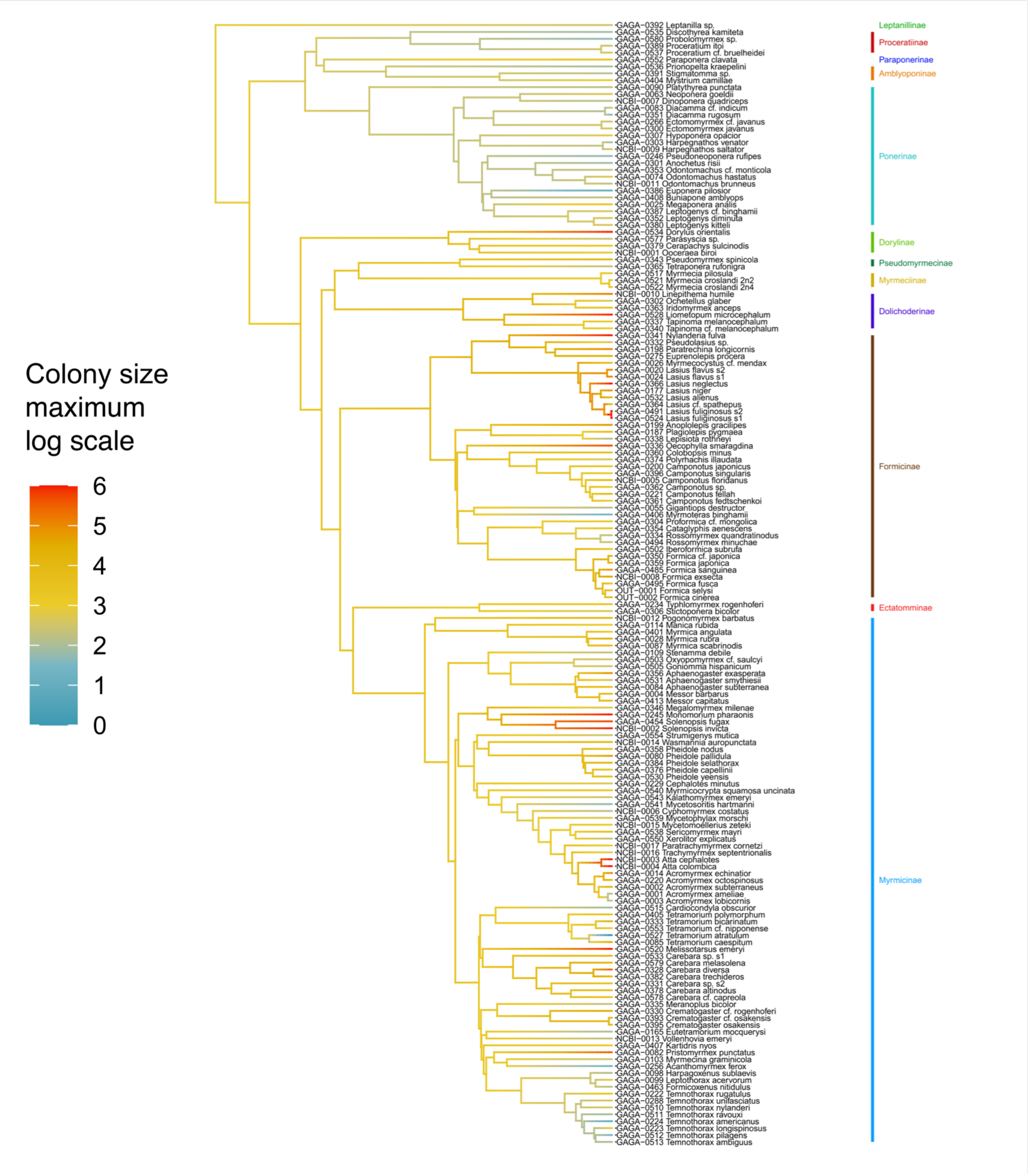

Figure S1: Ancestral state reconstruction of the log10 maximum colony size. Ancestral states were inferred using the R package *ape*.

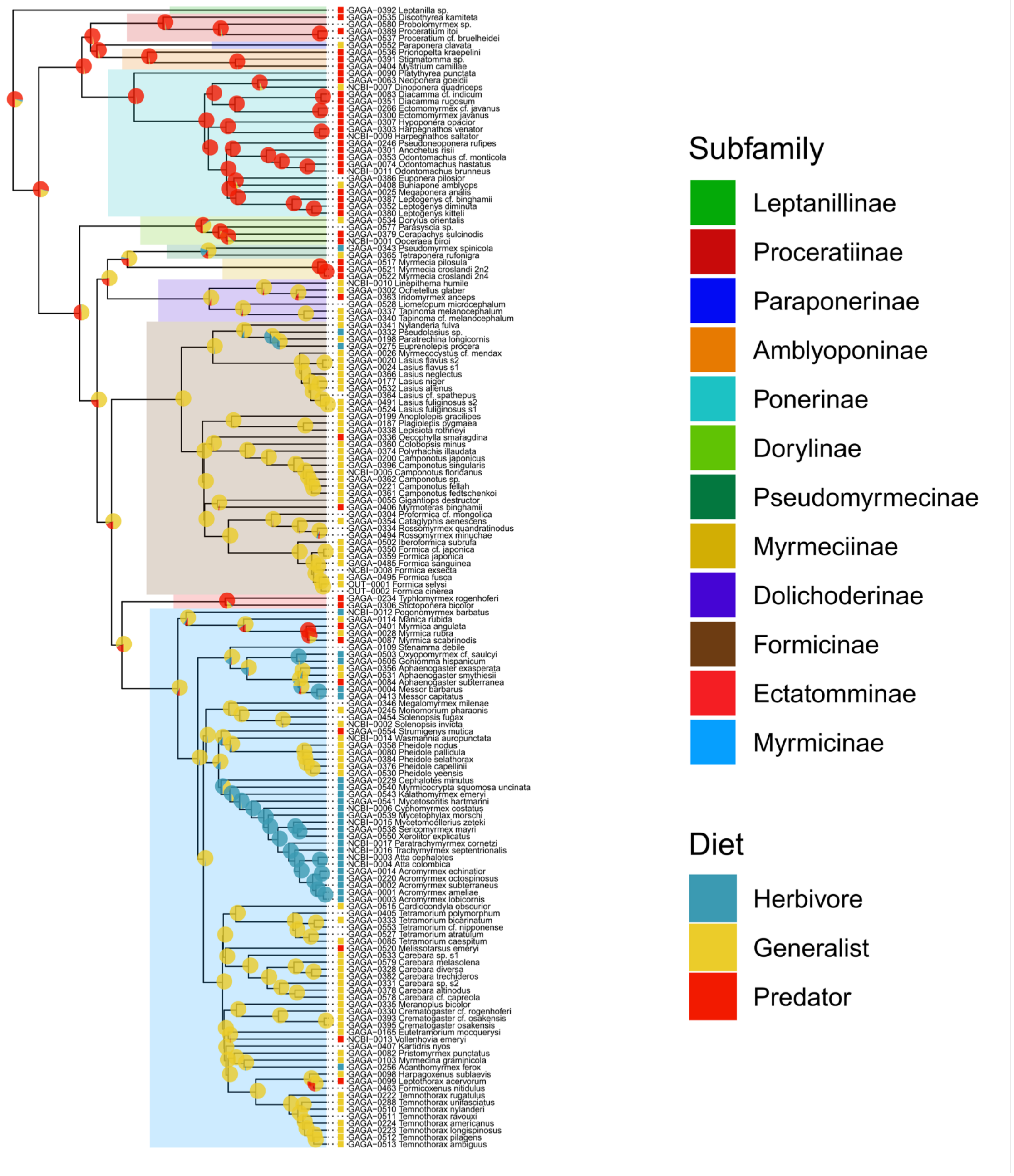

Figure S2: Ancestral state reconstruction of the diet. Ancestral states were inferred using the R package *ape*.

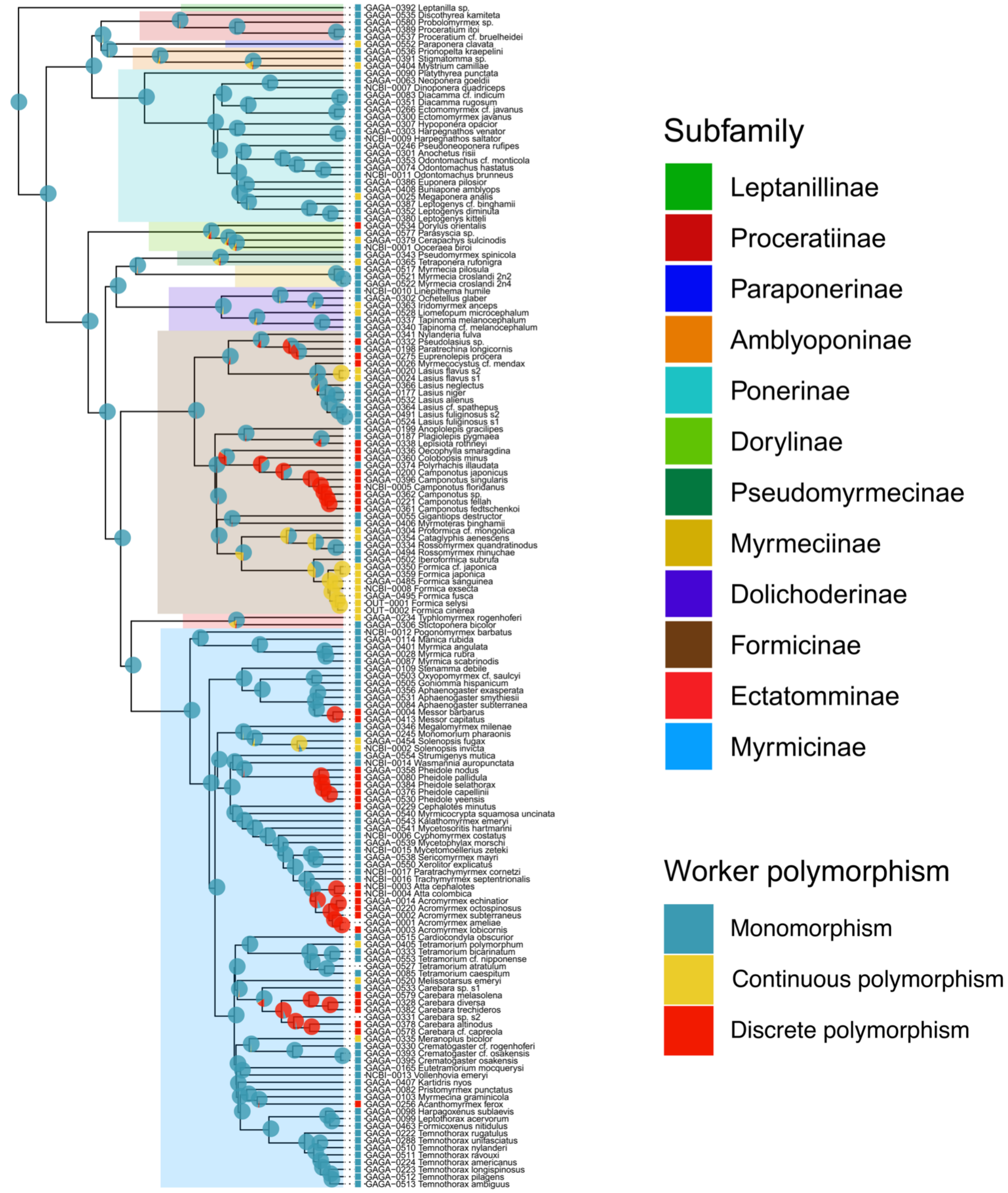

Figure S3: Ancestral state reconstruction of the worker polymorphism. Ancestral states were inferred using the R package *ape*.

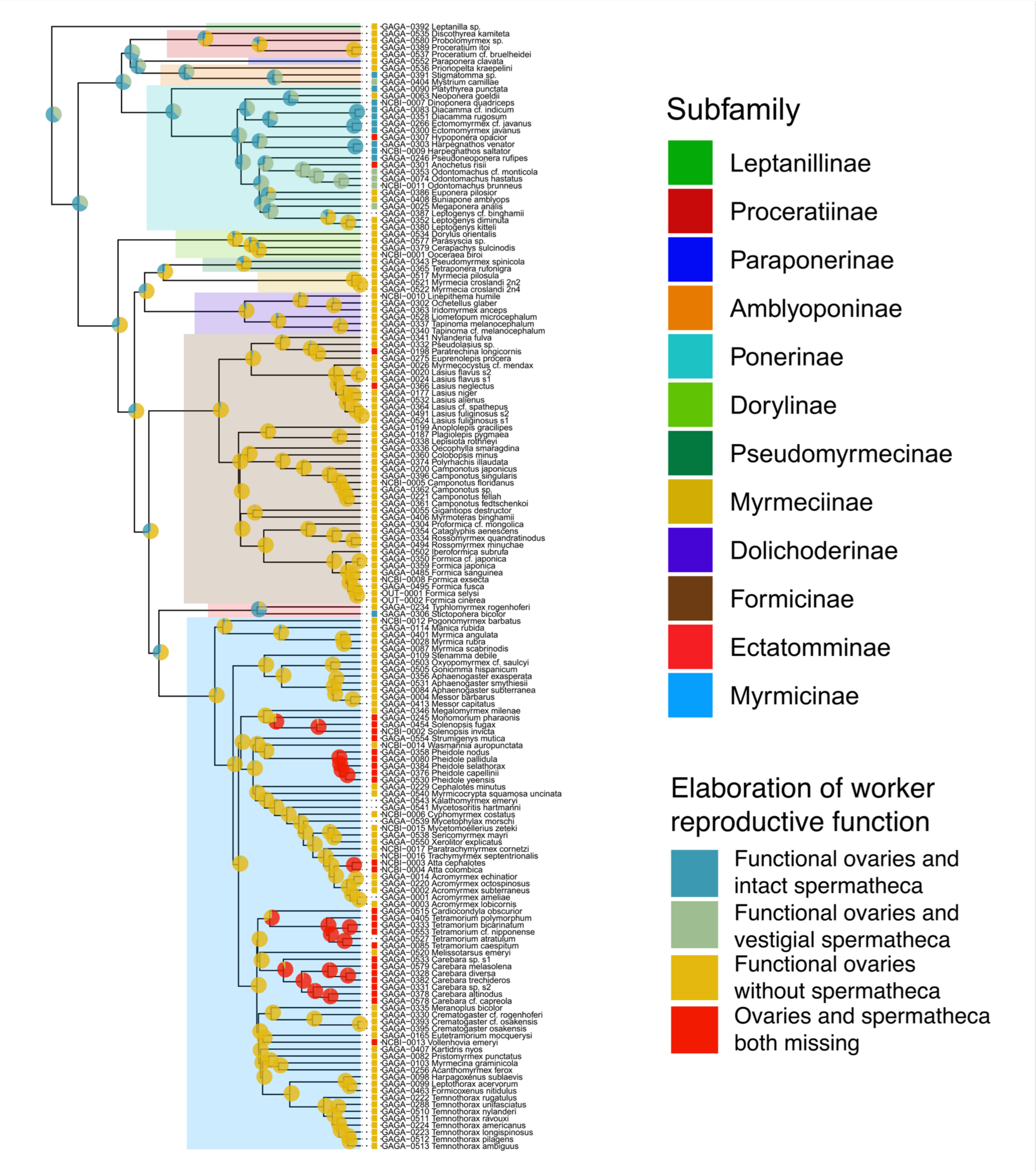

Figure S4: Ancestral state reconstruction of the worker reproductive function. Ancestral states were inferred using the R package *ape*.

Read Segmentation

Value	Analysis Metric
10,878,248	Reads
86,346,101	Segmented reads (S-reads)
1,500	Mean length of S-reads
97.49 %	Percent of reads with full arrays
7.94	Mean array size (concatenation factor)

S-read Length Distribution

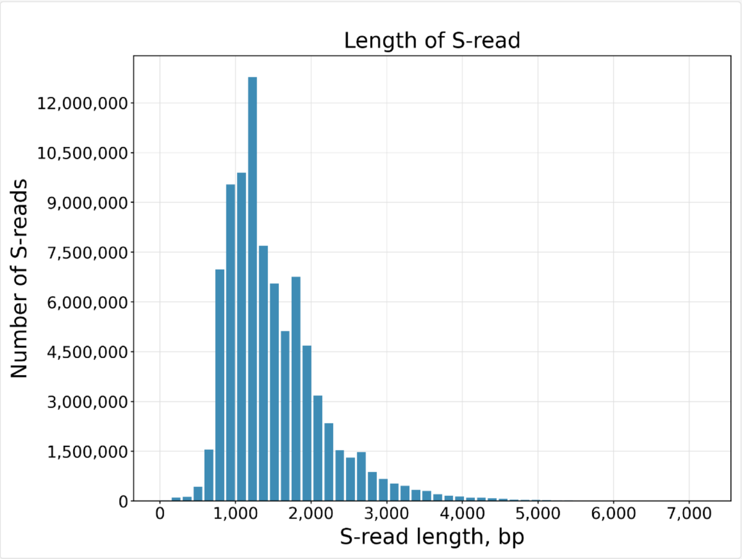

Figure S5: Overview of the Iso-Seq sequencing results.

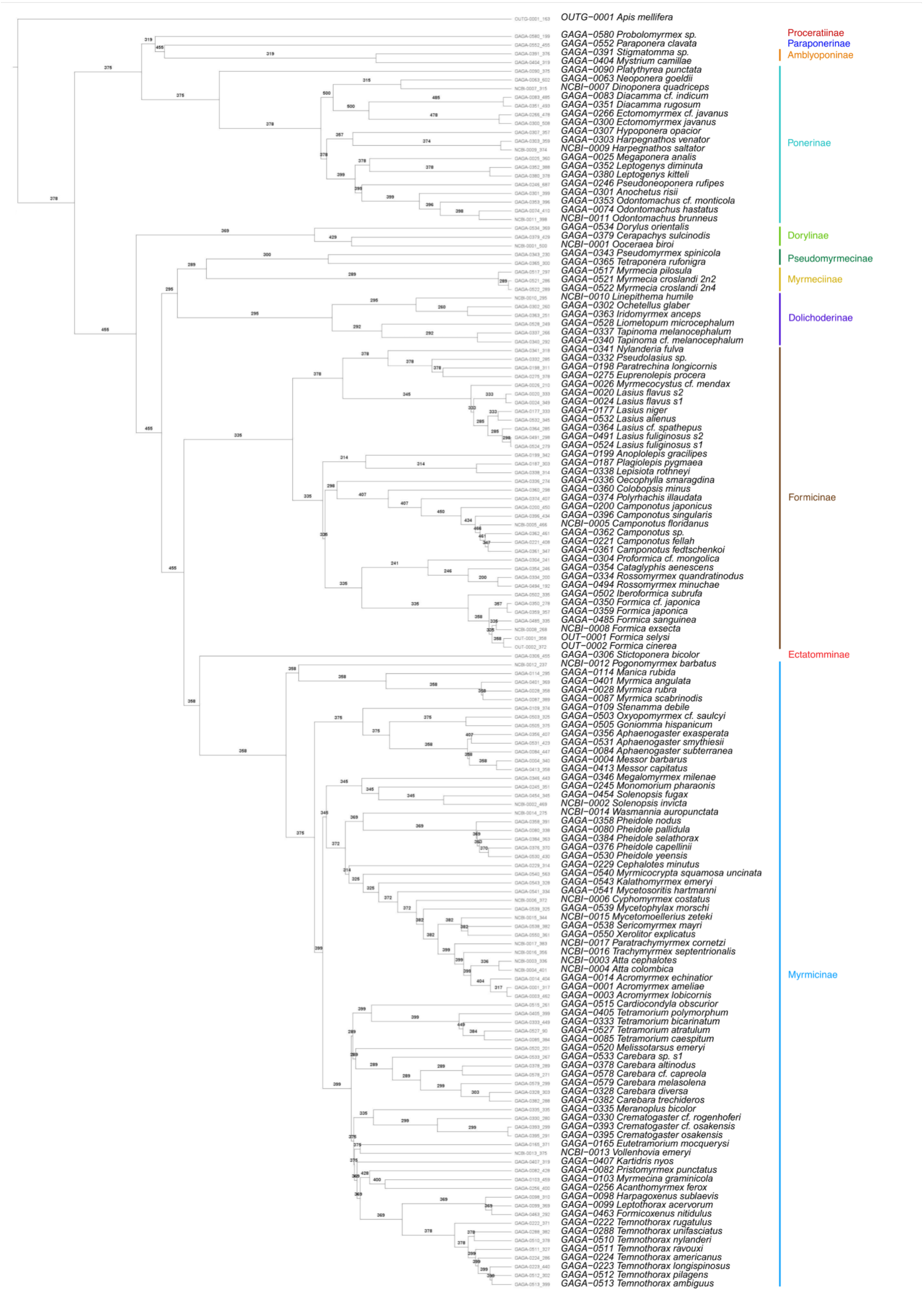

Figure S6: Inferred OR numbers plotted the ant phylogeny. The honey bee *Apis mellifera* is used as outgroup for all the ants.

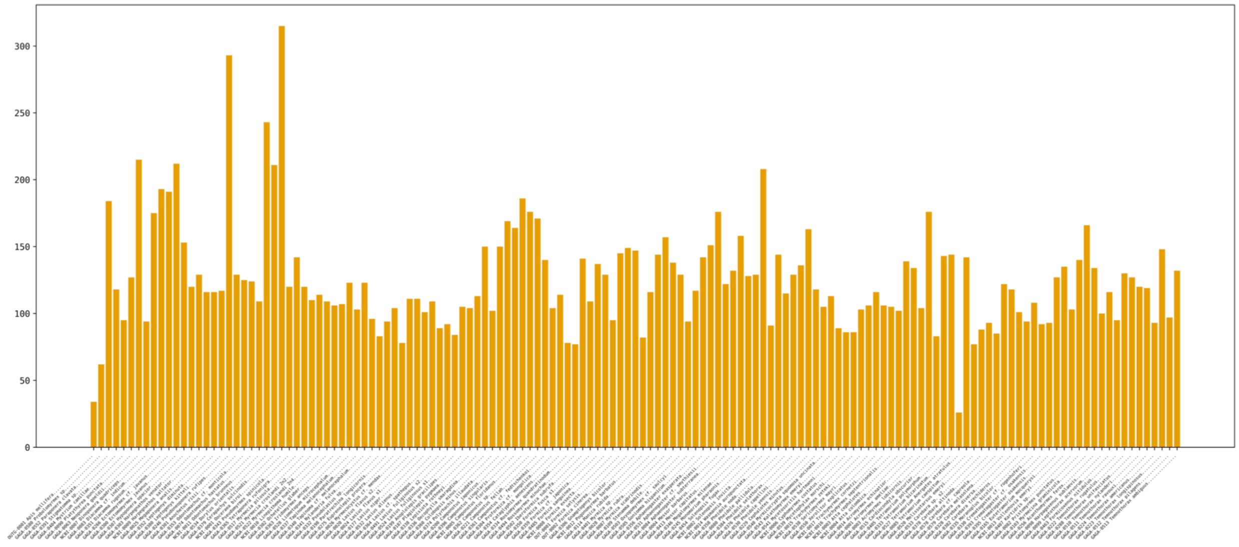

Figure S7: Number of subfamily 9-exon ORs per species

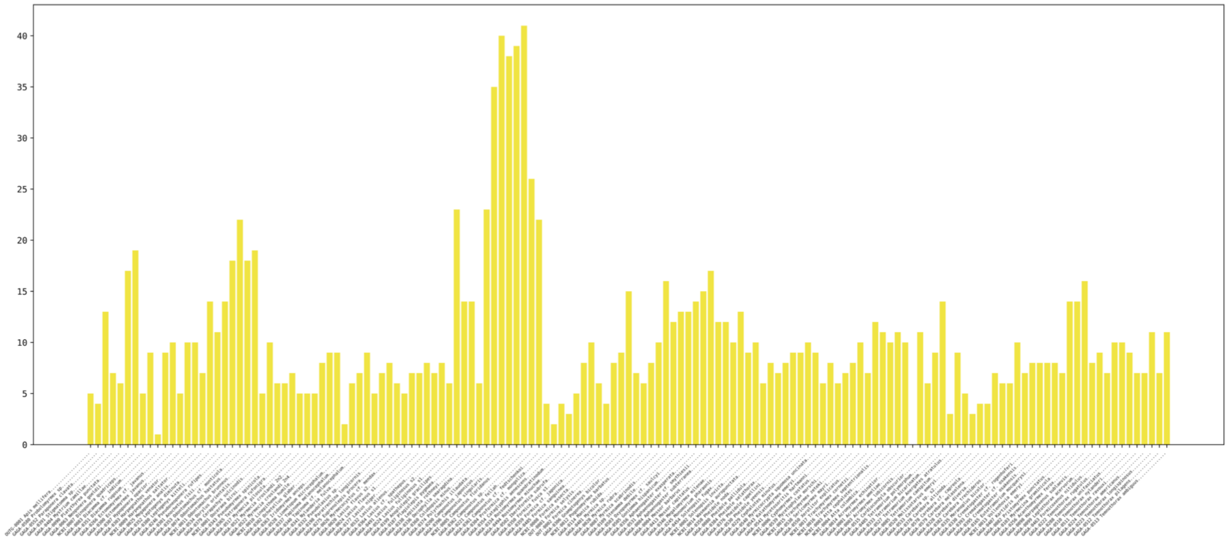

Figure S8: Number of subfamily P ORs per species

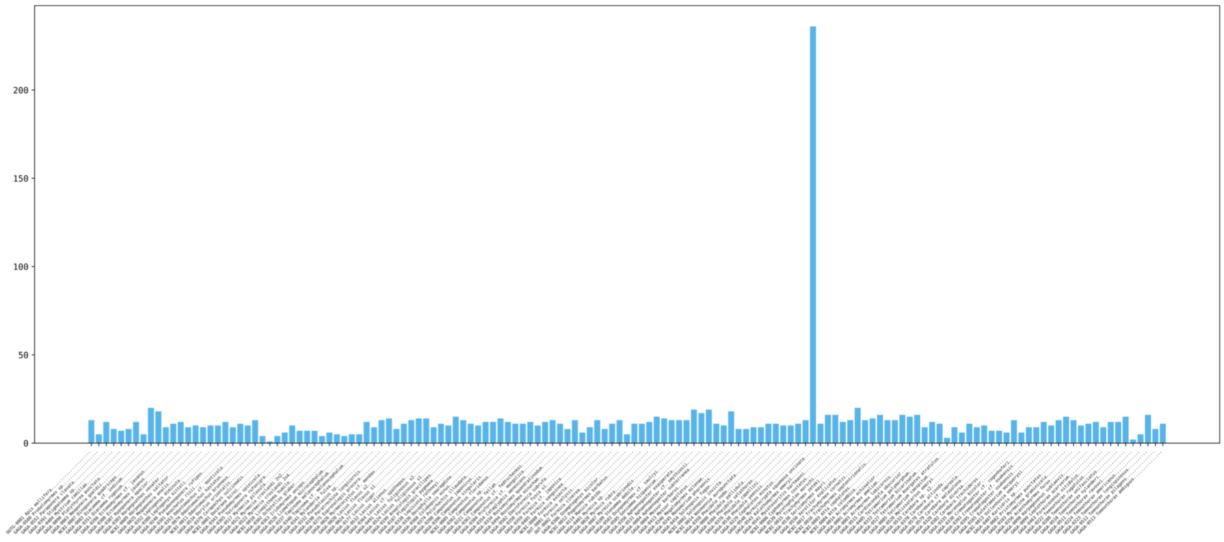

Figure S9: Number of subfamily H ORs per species

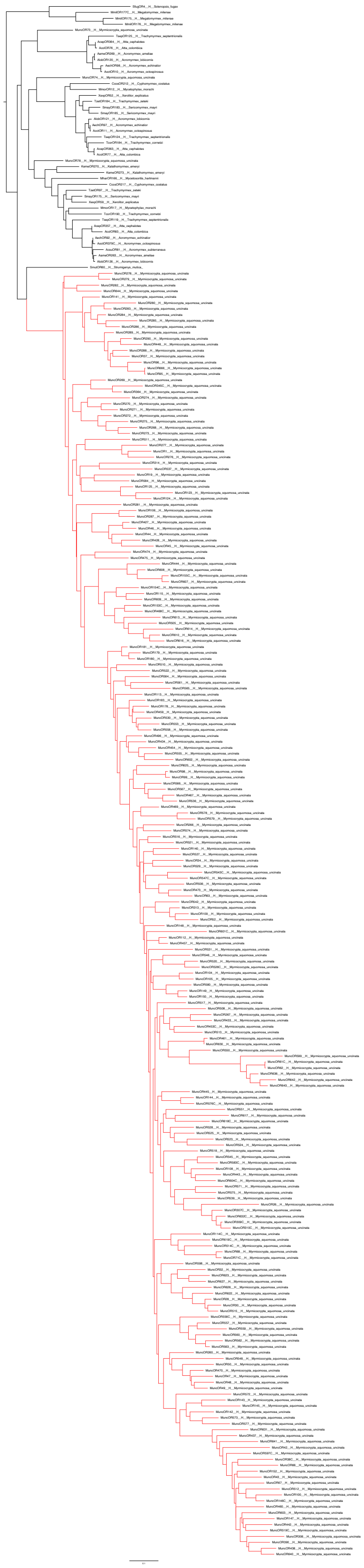

Figure S10: Phylogenetic tree showing the *Myrmicocrypta squomosa uncinata* specific expansion in the H ORs subfamily in red.
